# Hierarchical Stochastic Simulation Algorithm for SBML Models of Genetic Circuits

**DOI:** 10.3389/fbioe.2014.00055

**Published:** 2014-11-28

**Authors:** Leandro H. Watanabe, Chris J. Myers

**Affiliations:** ^1^Department of Electrical and Computer Engineering, The University of Utah, Salt Lake City, UT, USA

**Keywords:** hierarchical simulation, stochastic simulation, discrete-event simulation, SBML, genetic circuits, population modeling

## Abstract

This paper describes a hierarchical stochastic simulation algorithm, which has been implemented within iBioSim, a tool used to model, analyze, and visualize genetic circuits. Many biological analysis tools flatten out hierarchy before simulation, but there are many disadvantages associated with this approach. First, the memory required to represent the model can quickly expand in the process. Second, the flattening process is computationally expensive. Finally, when modeling a dynamic cellular population within iBioSim, inlining the hierarchy of the model is inefficient since models must grow dynamically over time. This paper discusses a new approach to handle hierarchy on the fly to make the tool faster and more memory-efficient. This approach yields significant performance improvements as compared to the former flat analysis method.

## 1 Introduction

Genetic engineering and other forms of biotechnology are having a substantial impact on the world economy, and this trend is likely to continue (Lucks and Arkin, 2011). Although genetic engineers have had success on the development of some pharmaceuticals, the field has encountered many challenges. One of the challenges is to gain control over the cells one needs to accurately and efficiently predict their behavior (Lucks and Arkin, 2011). This motivated the creation of the synthetic biology field, which is a subset of bioengineering developed by biologists and engineers. One of the goals of the field is the systematic design of *genetic circuits*. Genetic circuits are used to regulate gene expression at many molecular levels and can potentially be used to consume toxic spills and waste (Brazil et al., 1995; Cases and de Lorenzo, 2005), destroy tumors (Anderson et al., 2006; Ruder et al., 2011), and produce drugs or bio-fuels more efficiently (Ro et al., 2006; Savage et al., 2008).

Since *electronic design automation* (EDA) software tools have had success in the electrical engineering field in the construction of complex circuits, the synthetic biology field has introduced the development of *genetic design automation* (GDA) tools (Myers et al., 2009). iBioSim, being developed at the University of Utah, is one example of such a tool (Madsen et al., 2012a). The iBioSim tool can be used to model, analyze, and visualize genetic circuits. Models in iBioSim are represented using the *systems biology markup language* (SBML), which is a standard representation format for chemical reaction network models in systems biology (Hucka et al., 2003).

iBioSim has had positive results with static models (Kuwahara et al., 2010; Nguyen et al., 2010; Madsen et al., 2012a). However, many biological models are complex and rely on the communication and cooperation of cells. Recently, iBioSim has been extended to support such dynamic modeling of bacterial populations (Stevens and Myers, 2012). However, improvements in simulation performance are needed. Currently, the bottleneck in the analysis of population models in iBioSim is the routine that flattens out the hierarchical constructs. This routine is very expensive and causes the memory requirements of the model to grow quickly. This fact motivated the development of the *hierarchical stochastic simulation algorithm* (hSSA) described in this paper. This method handles hierarchy at runtime rather than compiling the hierarchical model into a flat model before simulation. An earlier version of the hSSA that supported only chemical *species* and *reactions* appeared in (Watanabe and Myers, 2014). This paper extends the hSSA to support all elements of SBML Level 3 Version 1, including *rules, events*, and *constraints*.

## 2 Genetic Circuit Models

All organisms are made up of cells. Some organisms are composed of a single cell (e.g., bacteria) and some are composed of many cells (e.g., humans). Within each cell, a *deoxyribonucleic acid* (DNA) molecule includes *coding sequences* (known as *genes*) that provide instructions on how to construct *proteins*. Proteins are macromolecules made from chains of amino acids that serve many important functions in all organisms. Protein synthesis begins with a process known as *transcription* in which an enzyme called *RNA polymerase* (RNAP) binds to a specific sequence in the DNA called a *promoter*. RNAP walks the DNA to produce a single-stranded *messenger RNA* (mRNA). The resulting mRNA sequence is converted into a sequence of amino acids by a *ribosome* using a process known as *translation*. This amino acid sequence then folds into a protein. The rate of this protein synthesis process can be regulated through the binding of proteins known as *transcription factors* to regions on the DNA called *operator sites*. That is, transcription factors can facilitate or block the binding of RNAP to certain promoters. The interaction of all these elements can be used to create networks that control the rate of transcription of the genes. These regulatory networks are known as genetic circuits.

**Figure 1:**
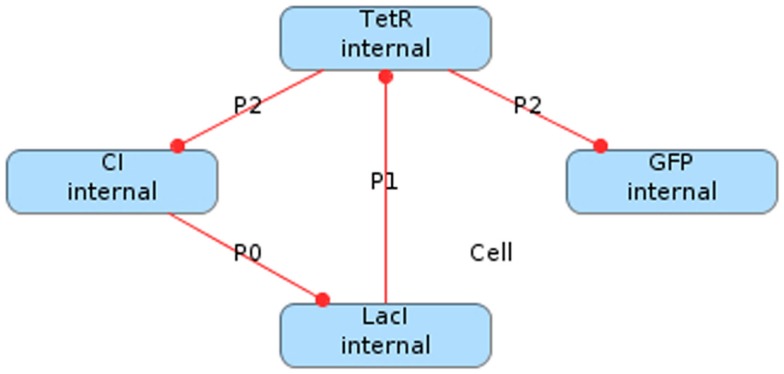
**Repressilator circuit modeled in iBioSim**.

One well-known genetic circuit is the *repressilator*, which was constructed in *Escherichia coli* (*E. coli*) (Elowitz and Leibler, 2000). In the repressilator, there are three proteins produced from three promoters in which each protein acts as a transcription factor for one promoter creating a loop that forms an oscillator. Namely, the first protein, LacI, inhibits the transcription of the production of the second, TetR, which inhibits the production of the third protein, CI, which inhibits the production of LacI. Figure 1 depicts a graphical model of this genetic circuit in iBioSim in which the vertices are proteins, the edges represent repression relationships, and the edge labels are the promoter names. Note that a fourth protein, *green fluorescent protein* (GFP), is included in this genetic circuit to be produced at the same time as CI. The purpose of this protein is to make the cells glow green when CI is high, allowing an observer to see the oscillation.

## 3 Chemical Reaction Models

**Figure 2:**
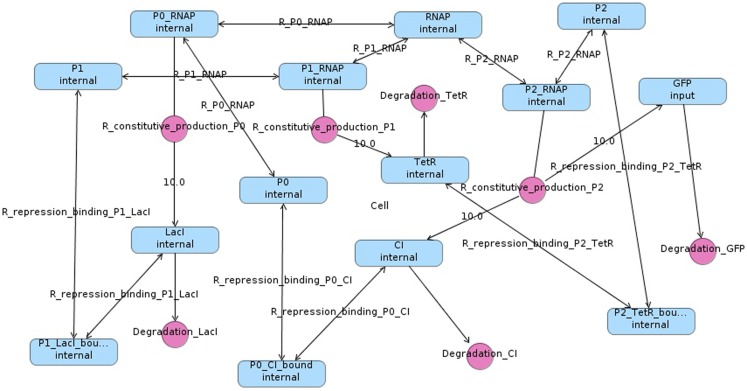
**Repressilator modeled as chemical reactions**.

In order to simulate the repressilator, the model must be converted into a set of *chemical reactions* (Myers, 2009). Chemical reactions combine *species* (DNA, RNA, protein molecules, etc.) to form new species. The species and chemical reactions for the repressilator circuit in Figure 1 are shown in Figure 2. Note that reactions can be shown explicitly as circles or implicitly as labels on edges between species. Also note that edges from species to reactions indicate that a species is a *reactant* (i.e., consumed by the reaction), edges from reactions to species indicate that a species is a *product* (i.e., produced by the reaction), and edges with no direction indicate that the species is a *modifier* (i.e., is neither produced or consumed). Finally, bi-directional edges indicate that a reaction is reversible, meaning that it can run in either direction. The number of molecules produced or consumed by a reaction is known as its *stoichiometry*. The edge is labeled with the stoichiometry when it is not one.

Some chemical reactions from the model are below:

(1) $$P0+\mathrm{RNAP}\overset{K_{o}}{↔}P0\text{\_}\mathrm{RNAP}\;\left(\mathrm{transcription initiation}\right)$$

(2) $$P0\text{\_}\mathrm{RNAP}\overset{k_{o}}{\to }P0\text{\_}\mathrm{RNAP}+10\cdot \mathrm{LacI}\;\left(\mathrm{production}\right)$$

(3) $$P1+2\cdot \mathrm{LacI}\overset{K_{r}}{↔}P1\text{\_}\mathrm{LacI}\text{\_}\mathrm{bound}\;\left(\mathrm{repression}\right)$$

(4) $$\mathrm{LacI}\overset{k_{d}}{\to }\left(\right)\;\left(\mathrm{degradation}\right)$$

Note that parameters such as *k*_0_ and *k_d_* are known as *rate constants*, and they indicate the speed or likelihood of the reaction. Parameters such as *K_r_* and *K_o_* are known as *equilibrium constants*, and they are ratios of the forward and reverse rate constants (i.e., *K_r_* = *k_rf_*/*k_rr_*).

## 4 ODE Simulation

A chemical reaction model can be converted into a set of ordinary differential equations (ODEs) using the *law of mass action*. This law states that the rate of a reaction is its rate constant times the concentration of the reactants raised to the power of their stoichiometry. More formally, consider a model with *n* species {*S*_1_, …, *S_n_*} and *m* reactions {*R*_1_, …, *R_m_*} where each reaction, *R_j_*, is of the form:

$$v_{1j}^{r}S_{1}+\ldots +v_{nj}^{r}S_{n}\overset{k_{f}}{\underset{k_{r}}{⇄}}v_{1j}^{p}S_{1}+\ldots +v_{nj}^{p}S_{n}$$

where $v_{ij}^{r}$ is the *reactant stoichiometry* for species *S_i_* in reaction *R_j_* and $v_{ij}^{p}$ is its *product stoichiometry*. Therefore, the law of mass action states that the *rate equation, V_j_*, for reaction *R_j_* is:

$$V_{j}=k_{f}\prod_{i=1}^{n}{\left[S_{i}\right]}^{v_{ij}^{r}}-k_{r}\prod_{i=1}^{n}{\left[S_{i}\right]}^{v_{ij}^{p}}$$

where [*S_i_*] is the concentration of species *S_i_*. The rate equations for all reactions that produce or consume a species, *S_i_*, can be combined to form an ODE describing the time evolution of the concentration of that species as follows:

$$\frac{d\left[S_{i}\right]}{\mathrm{dt}}=\sum_{j=1}^{m}v_{ij}V_{j},\;1\leq i\leq n$$

where $v_{ij}=v_{ij}^{p}-v_{ij}^{r}$ (i.e., the net change in species *S_i_* due to reaction *R_j_*). As an example, the ODE for LacI is as follows:

$$\begin{array}{llll} \frac{d\left[\mathrm{LacI}\right]}{\mathrm{dt}} & =10k_{o}\left[P0\text{\_}\mathrm{RNAP}\right]-k_{d}\left[\mathrm{LacI}\right]-2\left(k_{rf}\left[P1\right]{\left[\mathrm{LacI}\right]}^{2}\right.\; & & \;\\ & \;-\left.k_{rr}\left[P1\text{\_}\mathrm{LacI}\text{\_}\mathrm{bound}\right]\right)\; & & \; \end{array}$$

**Figure 3:**
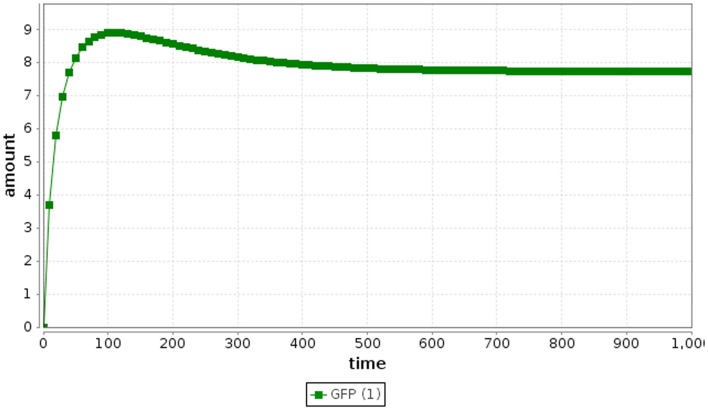
**Repressilator ODE simulation results**.

Ordinary differential equation simulation results for the repressilator are shown in Figure 3. It is clear from these results that ODE simulation of this model is not an accurate representation of the repressilator circuit, since the circuit stabilizes rather than oscillates. ODE simulation is deterministic, meaning that multiple simulations starting from the same initial condition always produce the same result. Moreover, ODE methods assume a large count of the entities being analyzed. In electrical engineering, ODE methods are reasonable for simulating electronic circuits, since the number of electrons flowing through the wires is very large. However, ODE methods can be inaccurate for certain genetic circuits, such as the repressilator circuit, because the numbers of molecules of each species in a genetic circuit are typically small discrete values (Kaern et al., 2005). In addition, since the number of molecules is typically quite small, the system can have large intrinsic noise making ODE methods less accurate. While there do exist ODE models that produce oscillations, our ODE model, which is directly derived from the chemical reaction network for this genetic circuit does not reproduce the expected oscillatory behavior. The original ODE model in (Elowitz and Leibler, 2000) has little physical connection to the biological behavior of the repressilator.

## 5 Stochastic Simulation

A better method for reasoning about genetic circuits is to utilize Gillespie’s *stochastic simulation algorithm* (SSA) (Gillespie, 1977). There are several variants of the SSA. This paper uses the *direct method*, which is shown in Algorithm 1. The SSA takes a chemical reaction network model, *M*, and computes a *time series simulation α*. The SSA is essentially a Monte Carlo algorithm, which treats each reaction as a random event. The simulation begins by initializing *α* to an empty sequence, computes the initial time and state, $\left\langle t,x\right\rangle$, from the model, *M*, and appends this *time point* to *α*. The state of the network is $x=\left\langle x_{1},\ldots ,x_{n}\right\rangle$ where *x_i_* is the current amount of species *S_i_*. The next step computes the *reaction propensities*, $a=\left\langle a_{1},\ldots ,a_{m}\right\rangle$, where *a_j_* is the propensity for reaction, *R_j_*, and can be approximated using the rate equation as follows:

$$a_{j}=k_{j}\prod_{i=0}^{n}{\left(x_{i}\right)}^{v_{ij}^{r}}$$

where *k_j_* is the rate constant for reaction *R_j_* and $v_{ij}^{r}$ is the number of reactant molecules of species *S_i_* consumed by the reaction. For example, the propensity for the forward reaction for transcription initiation on promoter P0 is approximately:

$$k_{of}\cdot P0\cdot \mathrm{RNAP}$$

**Table:** 

Algorithm 1: Gillespie’s SSA.
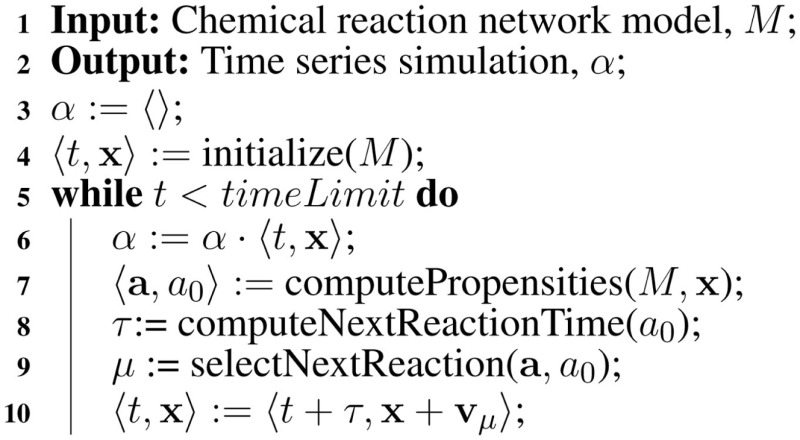

**Figure 4:**
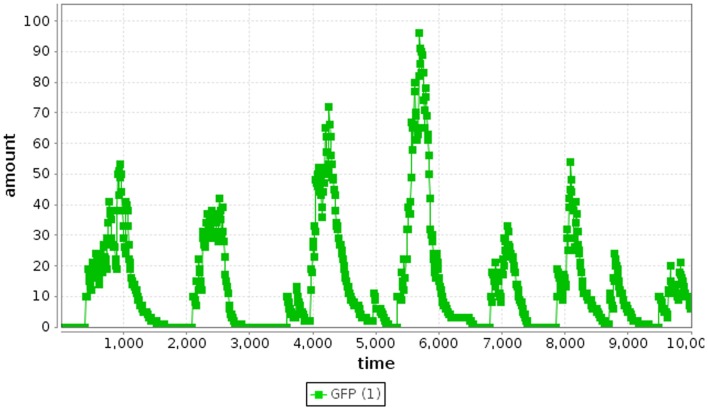
**Repressilator SSA simulation results**.

The total propensity, *a*_0_, is the sum of all propensities. The total propensity is used to the time until the next reaction using the following equation:

$$\tau =\frac{1}{a_{0}}\text{ln}\frac{1}{r_{1}}.$$

where *r*_1_ is a random number drawn from a uniform distribution from [0, 1]. Next, the propensities are used to compute the next reaction, *μ*, as follows:

$$\mu =\mathrm{smallest}\;\mathrm{integer}\;s\mathrm{.t}.\sum_{j=1}^{\mu }a_{j}>r_{2}a_{0}$$

where *r*_2_ is a random number drawn from a uniform distribution from [0, 1]. Finally, the time and the state are updated as shown in Algorithm 1, where **v**_μ_ is a vector representing the change in state due to reaction *R*_μ_. This process repeats until the time, *t*, exceeds the simulation time limit. Using the SSA method, the repressilator model indeed oscillates as shown in Figure 4.

## 6 Cellular Population Models

Genetic circuits have been constructed for many applications, such as genetic timers, oscillators, and logic gates, among others (Lucks and Arkin, 2011). These applications can be developed in single-celled organisms. However, there are applications in which population modeling is a necessity, such as biomedical applications (Ruder et al., 2011). For example, genetic circuits can potentially be used for the treatment of infectious diseases and cancer, vaccine development, and gene therapy.

**Figure 5:**
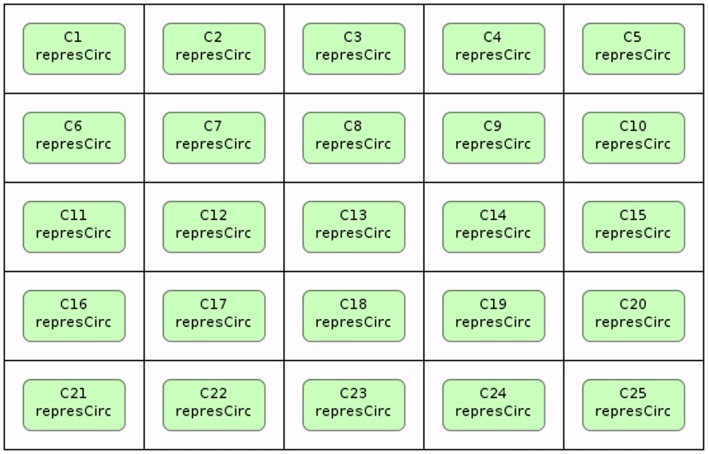
**Cellular population modeling in iBioSim**.

**Figure 6:**
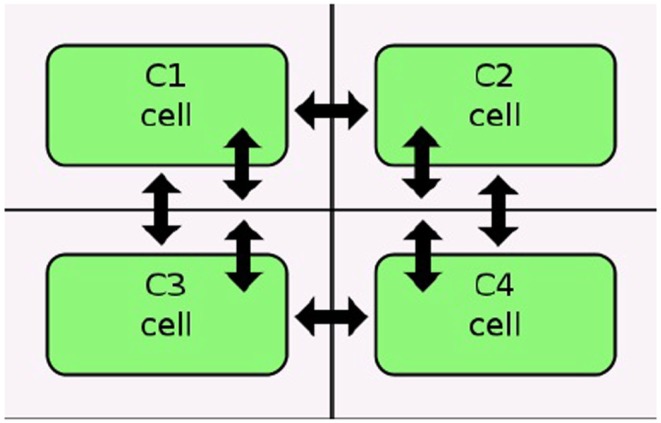
**Diffusion in an iBioSim grid model (courtesy ofStevens and Myers, 2012)**.

**Figure 7:**
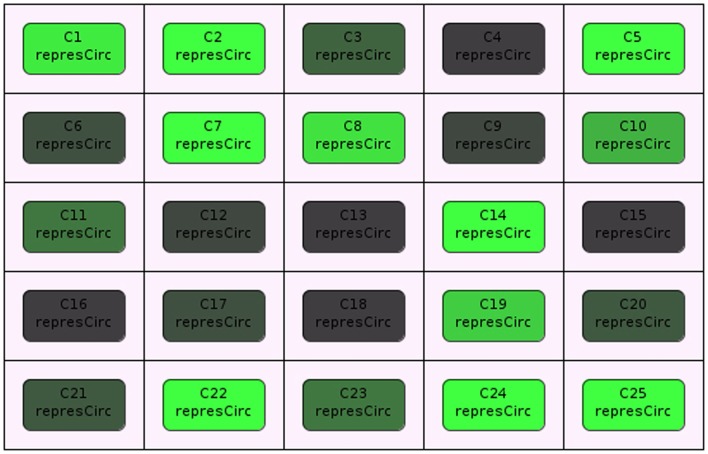
**Cellular population visualization in iBioSim**.

Stevens and Myers (2012) describe a method to model, analyze, and visualize dynamic populations of cells. Population-based models within iBioSim are represented in a two-dimensional grid as shown in Figure 5. Each grid location is distinct and each location can include a single cell. As shown in Figure 6, diffusible species within a cell can move through membranes and between neighboring grid locations. The simulator described in Stevens and Myers (2012) also supports dynamic events such as cell movement, division, and death. When a cell moves, the cell changes grid location. When a cell divides, a copy of the cell dividing is created. When a cell dies, the cell is removed from the model. Simulations can be visualized in iBioSim by coupling color gradients to species. The higher the concentration (or amount) of a species, the brighter the color. When visualizing the repressilator circuit, it is possible to see every cell periodically turning on and off over time as shown in Figure 7. While two dimensional grids are not as realistic as three dimensional models in biology, two dimensional grids are a useful simplification.

The hierarchy in grid models is represented using SBML’s hierarchical model composition package. This package allows the expression of hierarchy in SBML by allowing a top-level model to be constructed from a collection of sub-models. This package also enables the customization and connection of sub-models using *replacements* and *deletions*. A replacement can be used to state that an element in the top-level model replaces an element in a sub-model. A replacement can, for example, be used to state that a species in the top-level model is to replace a species in two sub-models, which effectively connects the two sub-models through this species. A deletion can be used to remove part of a sub-model that is not relavant to this use of the sub-model. A deletion, for example, can be used to remove a reaction that is not needed for this particular instantiation of a sub-model.

**Figure 8:**
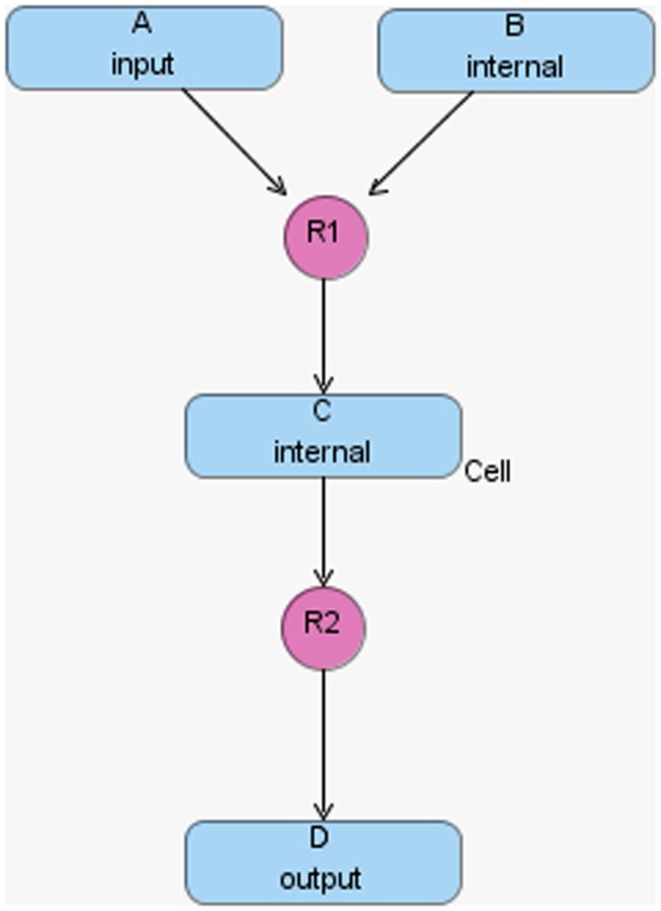
**Simple chemical reaction network**.

**Figure 9:**
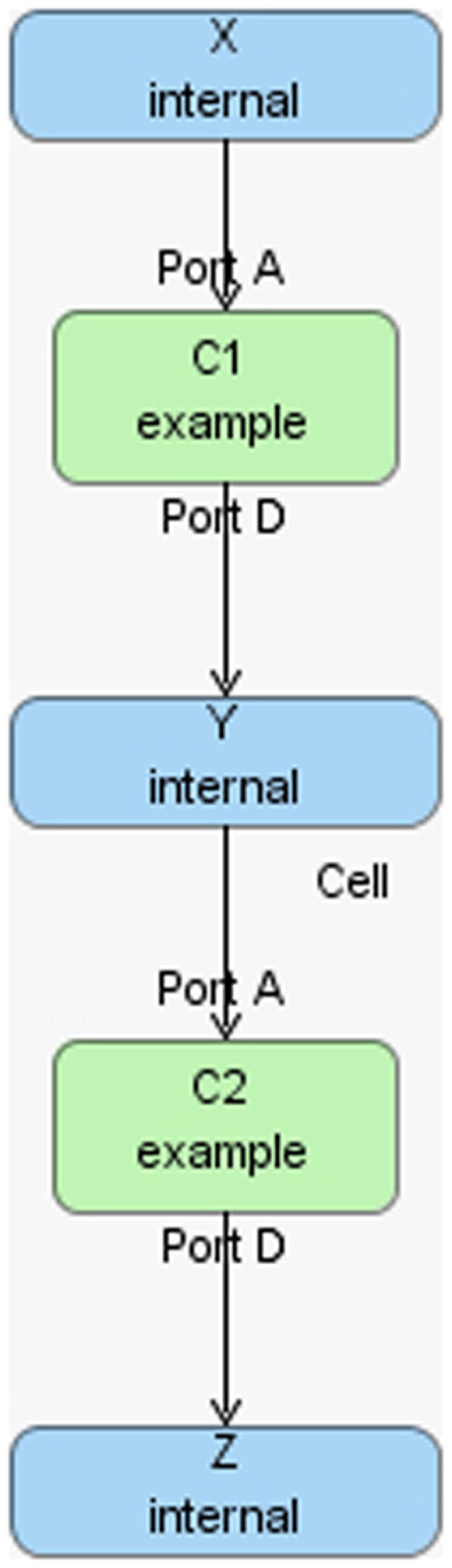
**Hierarchical model example**.

The hierarchical model composition package in SBML is better illustrated using an example. Assume there is a chemical reaction network as shown in Figure 8, where a molecule of A and B are taken as the reactants of a certain reaction R1 to form a molecule of C. In addition, a molecule of C is used to form a molecule of D through reaction R2. In this model, species A and D are put on a port, where the former is on an input port and the latter is on an output port. This chemical reaction network can be used to construct a hierarchical model as shown in Figure 9. In this model, the top-level model contains two instances, C1 and C2, of the chemical reaction network shown in Figure 8. In addition, the top-level model has three species X, Y, and Z, where species X replaces species A in instance C1, species Y is replaced by species D in C1 and replaces species A in C2, and species Z is replaced by species D in C2. Note that when a species in the top-level model replaces or is replaced by a species in a sub-model, the two species are effectively the same. Furthermore, reaction R2 in sub-model instance C2 is deleted from the respective model.

## 7 Hierarchical Simulation

Dealing with the hierarchy inherent in cellular population models can be difficult because there are many dependencies that need to be handled. Therefore, it is a common practice for many modeling tools to flatten (inline) the hierarchy of a model before simulation. In other words, a typical simulator would instantiate copies of each sub-model and perform replacements and deletions during this flattening process resulting in a potentially much larger model that no longer includes any hierarchical modeling constructs. This approach has several disadvantages. First, the flattening routine causes the size of the model representation to grow quickly, consuming a lot of computational resources. Second, the flattening process itself can be very time consuming. Using the simulator described in Stevens and Myers (2012), the time that iBioSim takes to flatten out a grid model composed of various numbers of repressilator components is shown in Table 1. According to this table, the time it takes to flatten out the hierarchy of a model can actually be larger than the time required to simulate the top-level model.

**Table 1:** **Comparison of flattening to simulation runtime**.

Grid size	Flattening (s)	Simulation (s)
2 × 2	1.101	0.488
4 × 4	9.482	2.458
6 × 6	40.065	8.408
8 × 8	119.619	24.272
10 × 10	285.714	62.709

This fact motivated the development of the hierarchical simulation method explained in Section 7.1. Section 7.2 illustrates the hierarchical method through an example. Section 7.3 presents extensions to the hSSA to support additional SBML constructs, such as, rules, events, and constraints.

### 7.1 hSSA overview

Our hierarchical simulator avoids the cost of flattening while preserving identical simulation results through several steps. First, in the preamble stage, the simulator locates the sub-models, {*M*_1_, …, *M_p_*}, used by the top-level model, *M*_0_. The simulator, however, only stores in memory one copy of each unique type of sub-model. The state of the simulator is now a vector of state vectors (i.e., $x=\left\langle x^{0},\ldots ,x^{p}\right\rangle$ where **x***^i^* is the state corresponding to model *M_i_*). Currently, the simulator only supports two-levels of hierarchy, so sub-models, which have sub-models are still flattened.

The SSA is modified as shown in Algorithm 2 to support hierarchical simulation. Structurally, the algorithms are similar. The main difference is the introduction of ν to indicate the model for the reaction to be executed. Since there is only one copy of each unique sub-model stored in memory, the key challenge is that replacements and deletions must be performed on the fly during simulation making each step a bit more involved.

**Table:** 

Algorithm 2: Hierarchical SSA.
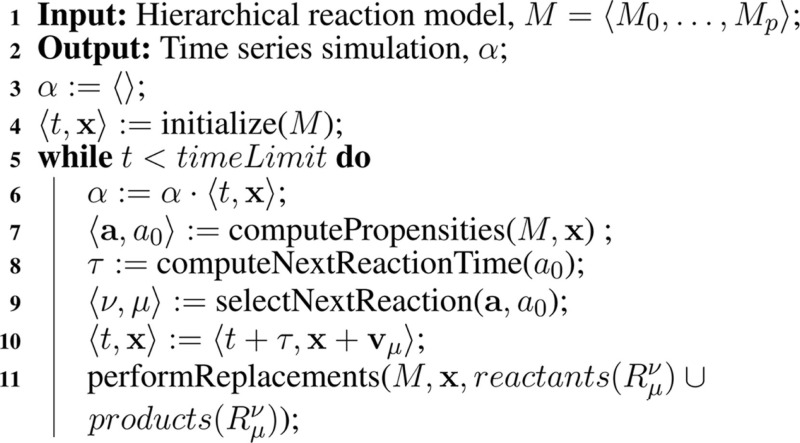

In the description of the algorithm below, the notation $replaces\left(S_{i}^{k},\;S_{j}^{l}\right)$ is used to indicate that species $S_{i}^{k}$ in model *M_k_* replaces species $S_{j}^{l}$ in model *M_l_*, and the notation $delete\left(R_{j}^{k}\right)$ indicates that reaction $R_{j}^{k}$ is to be deleted from model *M_k_*.

In the hSSA, replacements must be considered when determining the initial state, which is accomplished with Algorithm 3. First, the initial state vector is set to the initial value defined within each model. Next, each state in the top model, $x_{i}^{0}$ must be updated to take the value, $x_{j}^{k}$, of the initial state of a species $S_{j}^{k}$ when that species is specified to replace the top-level species $S_{i}^{0}$. Finally, the algorithm updates any species in a sub-model, which is replaced by a species at the top-level. These steps are necessary to ensure that the states of species involved in replacements coincide initially.

**Table:** 

Algorithm 3: initialize(*M*).
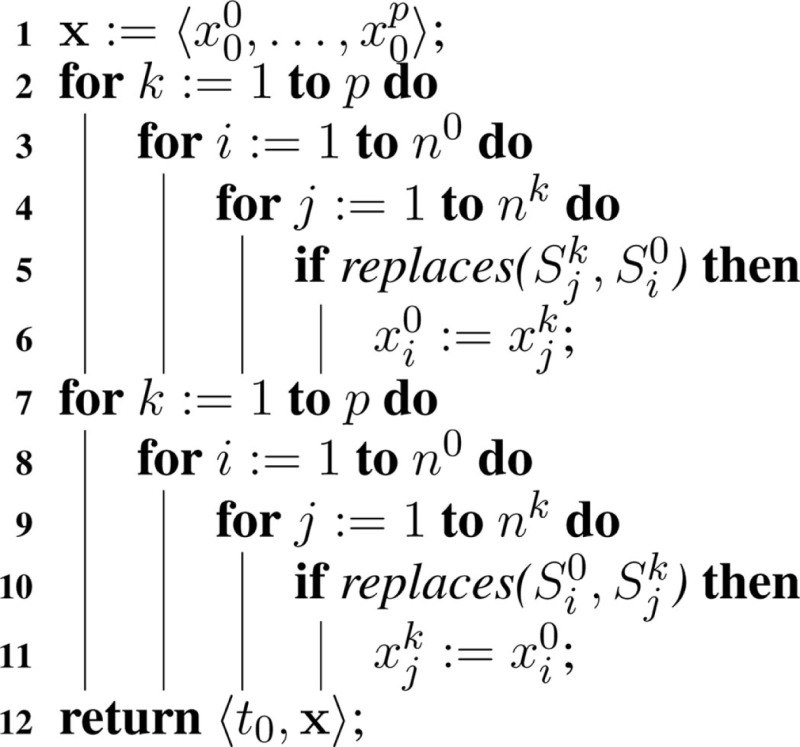

Deletions are considered when evaluating reaction propensities. Namely, in Algorithm 4, the propensity of a deleted reaction is set to zero, so it does not participate in the simulation. The total propensity calculated, *a*_0_, is the sum of the propensities for all the non-deleted reactions in all models.

**Table:** 

Algorithm 4: computePropensities(*M, x*).
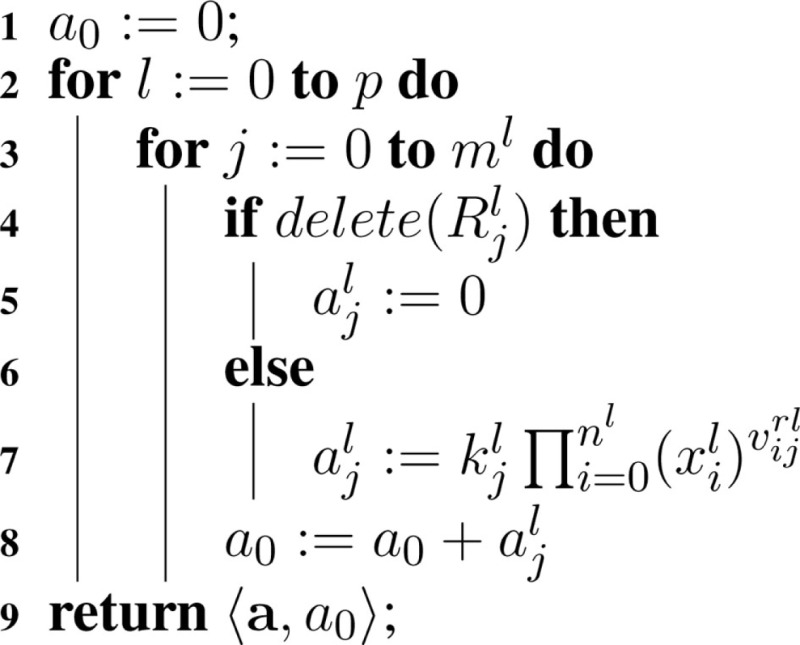

Computing the next reaction time is the same as for the original SSA, but the computation of the next reaction is modified as shown in Algorithm 5. Namely, the sum must be over all reactions in all models, and return both the model, *M_v_*, and the reaction, $R_{\mu }^{\nu }$, in this model to execute.

**Table:** 

Algorithm 5: selectNextReaction(a, *a*_0_).
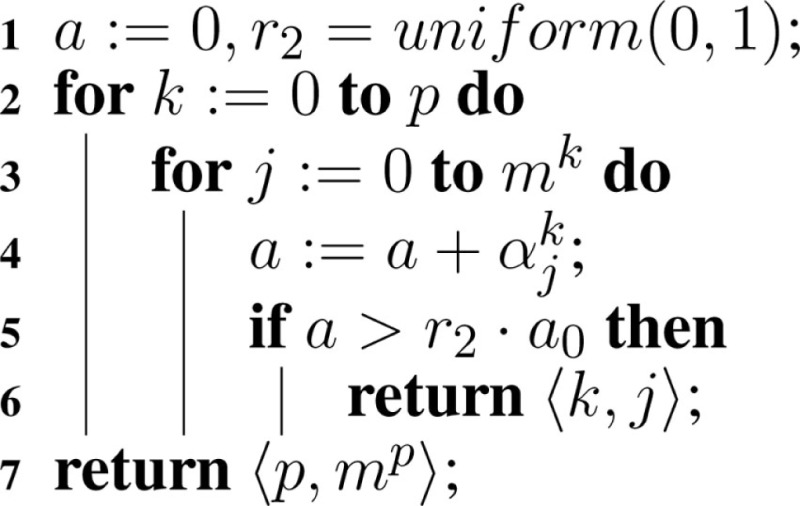

Finally, the current time is advanced to the next time step and the state of the model *M_v_* is updated as a result of the reaction $R_{\mu }^{\nu }$. The state of reactant and product species for this reaction that are involved in replacements must be updated in order to ensure that the values of these species continue to coincide throughout simulation. Namely, Algorithm 6 is passed a set of species, which have been updated. For each of these species, $y_{\delta }^{\mu }$, if this species is not from the top-level model (i.e., μ ≠ 0), then it must check if this species is involved in a replacement with a top-level species, $y_{i}^{0}$. If it is, this top-level species must be updated, and this algorithm must be called recursively to perform replacements on $y_{i}^{0}$. Otherwise, if this is a top-level species (i.e., μ = 0), then it must check if it is involved in a replacement for any species at a lower-level. If it is, then this species must be updated to take this value.

**Table:** 

Algorithm 6: performReplacements(*M*, x, *Y*).
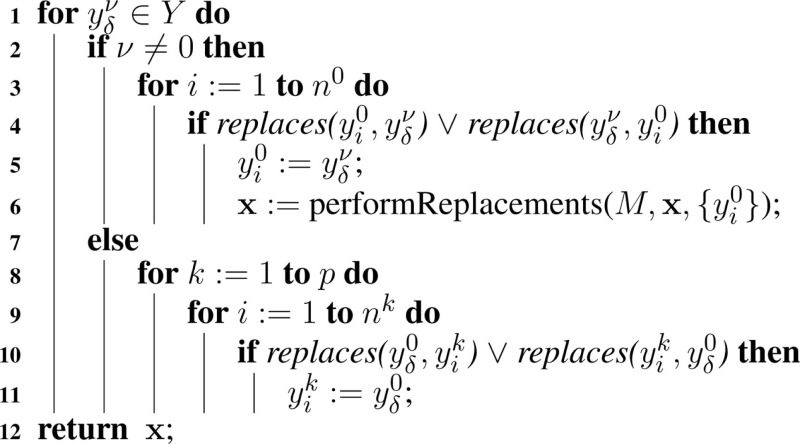

### 7.2 Example

To better illustrate how the algorithm works, the hierarchical model described in Section 6 is used as an example. In hSSA, the input is a collection of models where *M*_0_ represents the top-level model, and there are *p* sub-models. In this particular case, there are two sub-models where *M*_1_ represents instance C1 and *M*_2_ represents instance C2. The output is a times series simulation α, which is set to be initially empty. The first step in the initialization process is to set initial time to be equal to zero and the state vector **x** to contain the initial amount of each species in each model as shown in row Initial in Table 2. Note that the state vectors in C1 and C2 are equal since they refer to the same model definition. Once the state vectors are populated with the initial amount of each species, the simulator handles replacements. First, the simulator handles the case where a species in a sub-model replaces a species in the top-level model. Once this step is completed, the simulator is going to handle the case where a top-level species replaces species in sub-models. In the example, the value of species X perculates down to species A in instance C1. The same holds for the case where species Y replaces A in sub-model C2. Table 2 shows the final values once all replacements are handled.

**Table 2:** **The initial value of the state vector of the top-level model under top and the initial value of the state vector of the models C1 and C2 given in Figure 8, and how replacements affect the state vector of each model**.

	Top				C1					C2				
	t	X	Y	Z	t	A	B	C	D	t	A	B	C	D
Initial	0	5	10	10	0	10	10	0	0	0	10	10	0	0
Before	0	5	10	10	0	10	10	0	0	0	10	10	0	0
After	0	5	0	0	0	5	10	0	0	0	0	10	0	0

*In this particular model, species Y in the top-level model is replaced by species D in C1 and the value of Y is updated accordingly. Similarly, species Z in the top-level model is replaced by species D in C2. In addition, species X is replacing species A in C1 and species Y is replacing A in C2*.

After initialization is done, the simulator enters the loop. First, the simulator records the current state of the simulation. Then, the propensities for each reaction are calculated along with the total propensity as shown in Table 3. The next reaction and the next reaction time are computed afterward. From Table 3, it is possible to notice that the only possible reaction to be selected is reaction R1 in C1 given that it is the only reaction that has a propensity greater than zero. Table 4 shows the computed time for the next reaction to occur, as well as, the next reaction to be fired. The last step is to update the state of the simulation. Time is advanced to the next time step and the state vector **x** is updated based on the stoichiometry of the species involved in the selected reaction as shown in Table 5.

**Table 3:** **Propensity for each reaction and the total propensity, which is the sum of all reaction propensities**.

Reaction propensities				
C1		C2		Total
*a*_1_	*a*_2_	*a*_1_	*a*_2_	*a*_0_
5	0	0	0	5

**Table 4:** **The next reaction time is computed and the next reaction time is selected, which is a random variable drawn from an exponential distribution where the mean is the inverse of the total propensity**.

τ	ν	μ
0.1	C1	R1

*The next reaction to fire is selected based on the reaction propensities. That is, the next reaction is random with probability proportional to the contribution of this reaction’s propensity to the total propensity*.

**Table 5:** **Amount for all species in the hierarchical model after the first iteration**.

Top				C1					C2				
t	X	Y	Z	t	A	B	C	D	t	A	B	C	D
0	5	0	0	0	5	10	0	0	0	0	10	0	0
0.1	5	0	0	0.1	4	9	1	0	0.1	0	10	0	0
0.1	4	0	0	0.1	4	9	1	0	0.1	0	10	0	0

*Reaction R1 is selected, where a molecule of A and a molecule of B are consumed for the production of a molecule of C. Since species A in C1 is involved in a replacement, the new value of A needs to be percolated up to X in the top-level model. Now, the value of X is 4*.

These steps are repeated until the current time exceeds the time limit. Assuming the current time is still lower than the time limit, another iteration is performed. First, the current state of the simulation is recorded. Then, the propensities are computed as shown in Table 6. The next step is to compute the next reaction time, which, in this case, is 0.2. The next reaction selected is R2 in sub-model C1. Once the next reaction is selected, the state of the simulation is updated. Time is advanced to the next time step and the state vector **x** is updated after firing reaction R2 in C1. Table 7 shows the state after firing reaction R2 in C1 and handling replacements in the second iteration.

**Table 6:** **Propensity for each reaction for the second iteration, as well as, the total propensity**.

Propensities				
C1		C2		Total
*a*_1_	*a*_2_	*a*_1_	*a*_2_	*a*_0_
3.6	1	0	0	4.6

*Note that reaction R2 in sub-model C1 can be fired now, since a molecule of C is produced in the last iteration*.

**Table 7:** **Amount for all species in the hierarchical model after the second iteration**.

Top				C1					C2				
t	X	Y	Z	t	A	B	C	D	t	A	B	C	D
0	5	0	0	0	5	10	0	0	0	0	10	0	0
0.1	4	0	0	0.1	4	9	1	0	0.1	0	10	0	0
0.3	4	1	0	0.3	4	9	0	1	0.3	1	10	0	0

*The total amount of C is 0 and the amount of D is 1 in sub-model C1. The state update for species D is effectively affecting the state of Y in the top-level model, and consequently, species A in C2 since Y replaces A. In the update function, the value of D in C1 is percolated up to species Y in the top-level model and the value of Y is percolated down to species A in C2*.

After recording the state of the simulation, the propensities are calculated as shown in Table 8. Up until this point, C2 is unable to fire any reaction. However, species A in C2 has a molecule now, which enables reaction R1 to fire. The next reaction time that is drawn from an exponential distribution with mean 1/*a*_0_ is 0.2. The next reaction selected in this iteration is reaction R1 in C2. Once again, the state of the simulation is updated by advancing time to the next time step and the reaction is fired. The new state is shown in Table 9.

**Table 8:** **Propensity for each reaction and the total propensity for the third iteration**.

Propensities				
C1		C2		Total
*a*_1_	*a*_2_	*a*_1_	*a*_2_	*a*_0_
3.6	0	1	0	4.6

**Table 9:** **Amount for all species in the hierarchical model after the third iteration**.

Top				C1					C2				
t	X	Y	Z	t	A	B	C	D	t	A	B	C	D
0	5	0	0	0	5	10	0	0	0	0	10	0	0
0.1	4	0	0	0.1	4	9	1	0	0.1	0	10	0	0
0.3	4	1	0	0.3	4	9	0	1	0.3	1	10	0	0
0.5	4	0	0	0.5	4	9	0	0	0.5	0	9	1	0

*In this iteration, reaction R1 in C2 is selected and, in this reaction, a molecule of both species A and B is consumed for the production of a molecule of C. Since the amount of A is changed, the value of species Y in the top-level model and species D in C1 must be updated accordingly*.

Something interesting happens in the fourth iteration. After recording the state of the simulation, the propensities are calculated. Even though reaction R2 in C2 could, in theory, be fired since this reaction requires only a molecule of C, the propensity is zero as shown in Table 10. This is because the reaction is deleted, causing the reaction propensity to be always zero. That is, this reaction can never be fired. One final note, although in this example duplicate copies of local and top-level variables connected through replacements are shown, as a further memory saving optimization, our implementation only keeps one copy of these variables.

**Table 10:** **Propensity for each reaction and total propensity for the fourth iteration that illustrates deletion in hierarchical models**.

Propensities				
C1		C2		Total
*a*_1_	*a*_2_	*a*_1_	*a*_2_	*a*_0_
3.6	0	0	0	3.6

*When a reaction is deleted from the model, the propensity is always zero*.

### 7.3 Extensions to hSSA to support SBML

While the algorithm presented in Section 7.1 is limited to SBML models composed of only species and reactions, the actual implementation of our hierarchical simulator supports nearly all SBML Level 3 Version 1 core constructs, such as assignment rules, events, and constraints. The modifications necessary to support these are similar to those for reactions. Namely, deleted elements are dropped from sub-models, math expressions are computed on local states, and care must be taken to ensure that top-level model and local sub-model states for variables involved in replacements must always coincide throughout simulation. Algorithm 7 shows how these features can be incorporated into hSSA, and the rest of this section describes the modifications in more detail.

**Table:** 

Algorithm 7: Extended Hierarchical SSA.
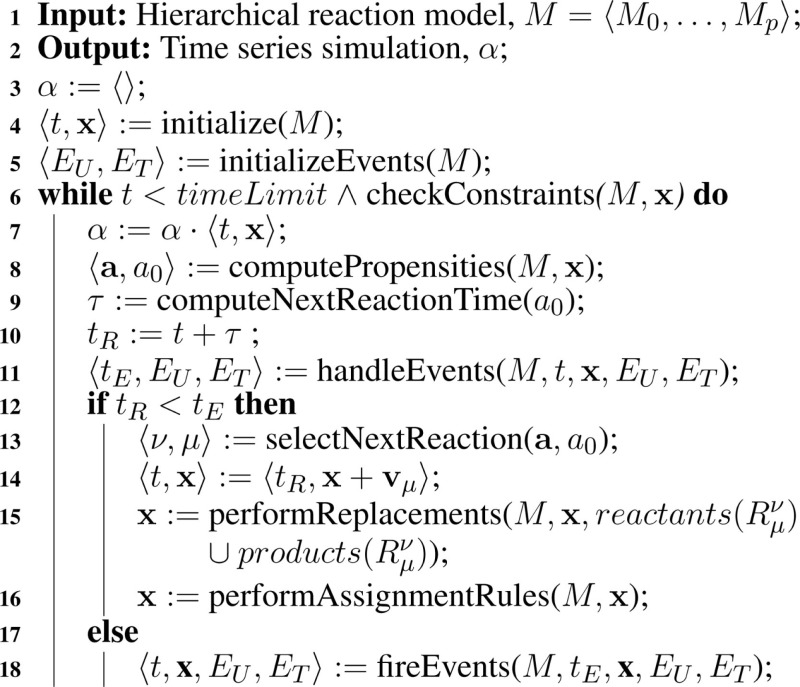

In core SBML, there are five types of objects that can take a value: compartments, species, parameters, species references, and reactions. Algorithm 7 extends the state vector, **x**, to take values of all of these types. Elements can have an initial assignment or be involved in an assignment rule that changes the value at the starting point of simulation. Thus, Algorithm 3 takes into account whether a variable’s value is determined by one of these. If so, the math is evaluated and the initial value of the object is updated accordingly. Additional SBML constructs that are not described are fast reactions, delay functions, algebraic rules, and rate rules.

The extended hSSA supports assignment rules, where a variable’s value is associated with a math function. The algorithm for performing assignment rules is shown in Algorithm 8. This algorithm goes through each assignment rule, *AR*, in each model. In this function, if the assignment rule *AR* in the model *i* for object *j* exists, and the assignment rule is not deleted, then the math associated with the rule is evaluated and the state vector **x** gets updated. Since the variable associated with the rule can participate in a replacement, replacements for this particular variable must be performed. Since assignment rules can affect the math of other rules, they need to be evaluated until there is no change in the evaluations.

**Table:** 

Algorithm 8: performAssignmentRules(*M*, x).
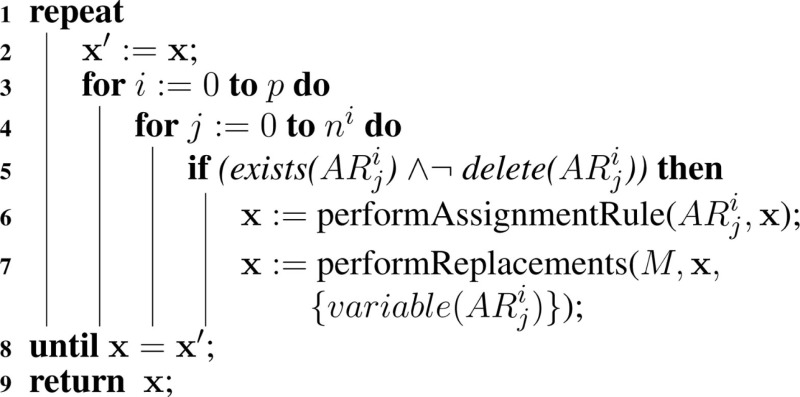

Another extension to the hSSA algorithm is the support for constraints, which are terminating conditions to the simulation. In each model, there are *c* constraints in a set of constraints, *C*. Simulation ends if any constraint in *C* evaluates to false. At the beginning of each iteration, hSSA evaluates all of the constraints in each model that are not deleted using the function illustrated in Algorithm 9.

**Table:** 

Algorithm 9: checkConstraints(*M*, x).
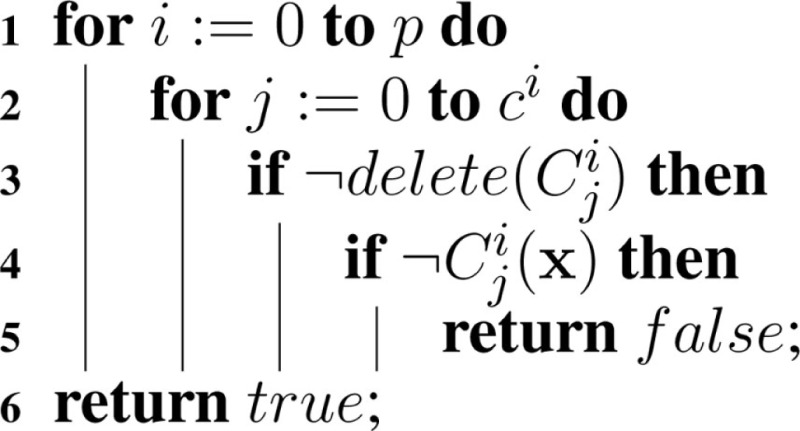

SBML models include a powerful discrete-event formalism, which adds much of the complexity to Algorithm 7. To support events, Algorithm 10, Algorithm 11, and Algorithm 12 are added. Two sets are introduced in these algorithms: *E_U_* and *E_T_*. The untriggered events are stored in the set *E_U_* and the triggered events are stored in the set *E_T_*. These sets are initialized using Algorithm 10. Each model *i* has *e^i^* events, $E_{j}^{i}$. Each event is analyzed during the initialization process. If an event is deleted, the event is not evaluated since deletion on events prevents them from ever being fired. However, non-deleted events require their initial condition or trigger condition to be evaluated, where the initial condition of a certain event $E_{j}^{i}$ is evaluated using *triggerInitial*$\left(E_{j}^{i}\right)$ and the trigger condition is evaluated using *trigger*$\left(E_{j}^{i}\right)$. All events that are initially false or the trigger condition is evaluated to false are inserted into *E_U_*.

**Table:** 

Algorithm 10: initializeEvents(*M*).
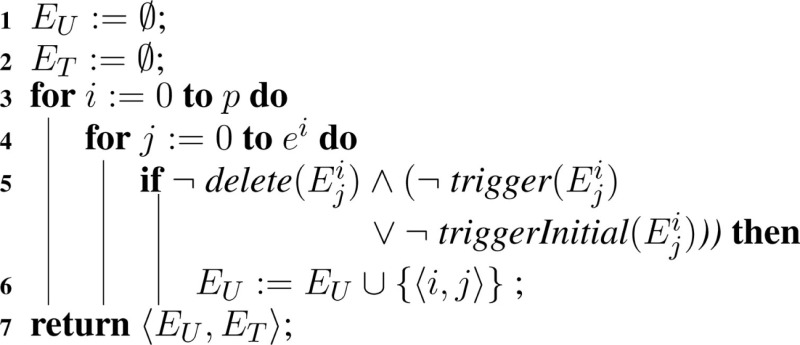

**Table:** 

Algorithm 11: handleEvents(*M*, *t*, x, *E_U_, E_T_*).
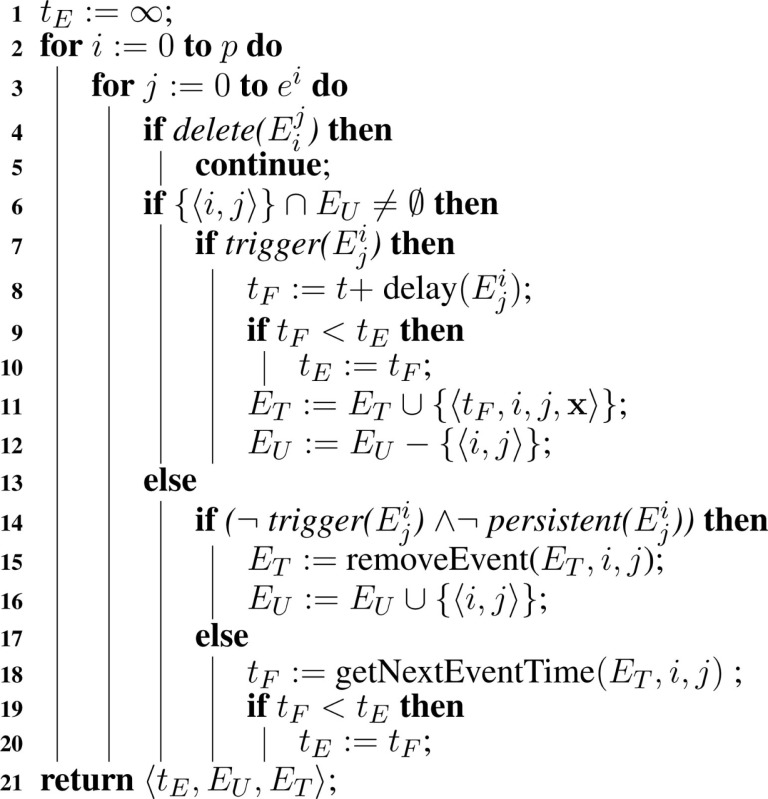

**Table:** 

Algorithm 12: fireEvents(*M*, x, *t_E_, E_U_, E_T_*).
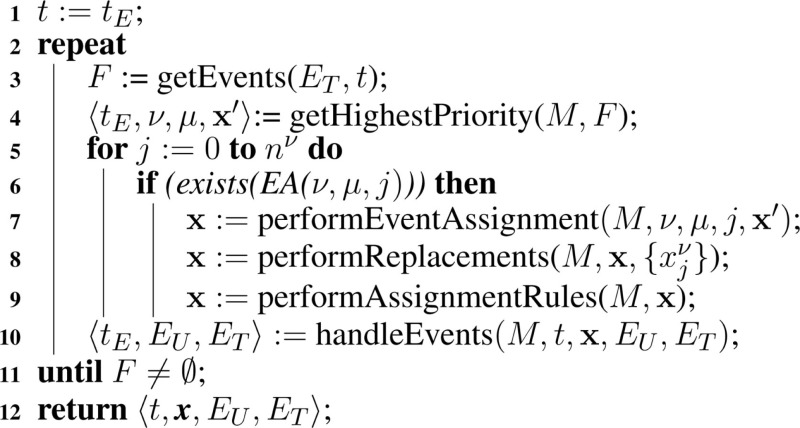

After initializing the model and the event sets, the hSSA needs to handle events using Algorithm 11. The algorithm keeps track of *t_E_*, the time of the next event scheduled to fire. Algorithm 11 loops through each event, if event $\left(E_{j}^{i}\right)$ is deleted, then the algorithm does nothing. Otherwise, the algorithm checks if the event becomes enabled and ready to fire. An event is scheduled to fire when its trigger condition is evaluated to true and the trigger condition previously evaluated to false. Thus, the function checks if event $\left(E_{j}^{i}\right)$ exists in *E_U_*, since the set contains events that previously evaluated to false. If event $\left(E_{j}^{i}\right)$ is in *E_U_*, then the trigger condition is evaluated. If the event is triggered, then the time *t_F_* in which the event is going to be fired is calculated, where *t_F_* is the current time, *t*, plus the delay associated with the particular event, where the delay is evaluated using *delay*$\left(E_{j}^{i}\right)$. If this event is scheduled to take place before the earliest event previously scheduled, then the next event time *t_E_* is updated and set to be equal to *t_F_*. The event is added to the triggered events set *E_T_* along with the time the event is supposed to fire and the current state of the simulation. The state vector **x** is needed because events can use the values from trigger time. The event must also be removed from *E_U_*. On the other hand, if the event $\left(E_{j}^{i}\right)$ is not in *E_U_*, then the event needs to be evaluated again and checked if the event is still allowed to fire. That is, if the event trigger is evaluated to false and the event is not persistent, given by *persistent*$\left(E_{j}^{i}\right)$, then all instances of this event in the set *E_T_* are removed from the set and the event is added to the set *E_U_*. If the event is enabled, then the firing time of this event is retrieved using *getNextEventTime*(*E_T_, i, j*). If this event is scheduled to happen before the current scheduled event, then the time of the next event gets updated to this event’s firing time to reflect the fact that this event takes precedence over the other evaluated events.

After handling the events, the algorithm needs to decide whether the next action is to fire a reaction or an event. In order to do so, the algorithm needs to keep track of two additional times: *t_R_* and *t_E_*. The former indicates the time of the next reaction and the latter indicates the time of the next event, and whichever is scheduled to happen first takes precedence over the other. If there is a reaction preceding the events, then the algorithm performs the same way as in Algorithm 2 in Section 7.1. The only difference is that the extended hSSA supports assignment rules, where a variable’s value is associated with a math function. After firing the reaction, replacements must be performed, followed by assignment rules that need to be evaluated due to the change in the state of the variables caused by the reaction.

If, on the other hand, there is an event scheduled to take place before the next reaction, all events preceding the next reaction are fired using Algorithm 12. In this algorithm, the current time is advanced to the next event time. Then, all events that are enabled are retrieved using *getEvents*, which returns a set of all events that are scheduled to fire at *t_E_*, and this set is assigned to set *F*, which is a set local to the *fireEvents* function that keeps track of the events that are ready to fire. The next event to fire is selected from *F* using the function *getHighestPriority*, which selects event μ in model ν based on the priorities of the events scheduled to fire. This function also returns the state vector **x**′, which is the state of the simulation when the event was triggered. For each object in model ν, there is a check if the selected event has an event assignment for object $x_{j}^{\nu }$. If it does, then the math associated with the event assignment is evaluated and **x** gets updated accordingly. Since the variable involved in the event assignment can be involved in a replacement, replacements must be performed to maintain consistency of the objects. Assignment rules are performed afterward, since the update in **x** can cause a change in the assignment rules’ math function. After all the assignments rules are performed, events need to be handled again since an event assignment or assignment rule can trigger a new event.

## 8 Results

**Figure 10:**
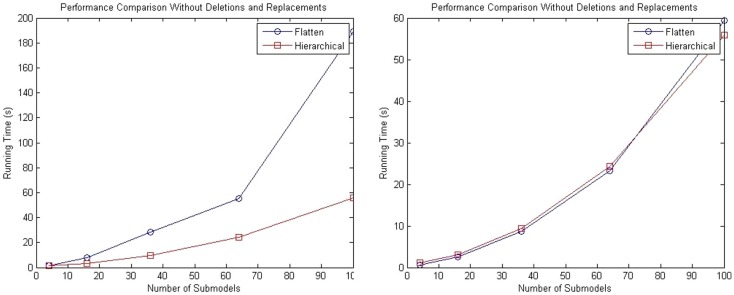
**Comparison of performance of SSA using flattening and our hierarchical approach**. The plot on the left shows the total running time for both approaches, and the plot on the right shows only the running time for both approaches without taking into consideration the time to flatten out the hierarchy.

While the complexity of the algorithm from a theoretical standpoint has not changed, the hSSA provides substantial improvements in performance relative to flat simulation methods. The hierarchical simulator performance is compared against the SSA simulator in Stevens and Myers (2012). Tests are performed using an Intel (R) Core (TM) i5 CPU 2.80 GHz and 4 GB RAM. The first test consists of a top-level grid model that is populated with repressilator sub-models without replacements or deletions. The test is performed using 4, 16, 36, 64, and 100 sub-models, and the total runtime results are shown in the left plot of Figure 10. The increase in runtime for hierarchical simulation is clearly more scalable than for flat simulation. The plot on the right in Figure 10 shows the results in simulation time using both approaches. It is possible to observe that hSSA performed almost equally compared to the simulation that flattens out the hierarchy and better for a 10 × 10 grid model, since the hSSA deals with smaller data structures.

A second test is performed using a top-level model populated with repressilator circuits in which the degradation reaction of the GFP reporter protein is deleted from all sub-models, and the GFP protein is replaced by a top-level GFP protein that tracks the total amount across all sub-models.

**Figure 11:**
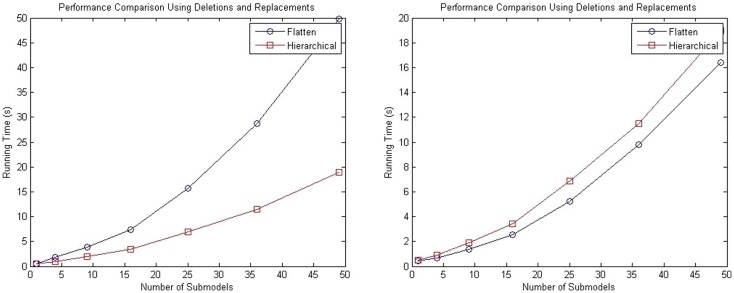
**Comparison of performance of the SSA using flattening and our hierarchical approach for a model that includes replacements and deletions**. The plot on the left shows the total running time for both approaches, and the plot on the right shows only the running time for both approaches without taking into consideration the time to flatten out the hierarchy.

Performance tests are performed using 1, 4, 9, 15, 25, and 50 sub-models, and the total runtime results are shown in the left plot of Figure 11. These results show that even with the added complexity of replacements and deletions, the performance of the hierarchical simulator still scales much better than an SSA simulator that uses flattening. The plot on the right of Figure 11 shows the run time results of only simulation using both approaches for the test where the connection of sub-models are customized using replacements and deletions. Note that these results are for single runs. It is possible to observe that hSSA has some overhead, which implies that, for a sufficient large time limit, the time required to simulate SSA with flattening and hSSA will intersect, and thus, the flattening algorithm is compensated over a long run or multiple runs. However, for our application, we are interested in shorter individual runs for visualization of population dynamics.

**Figure 12:**
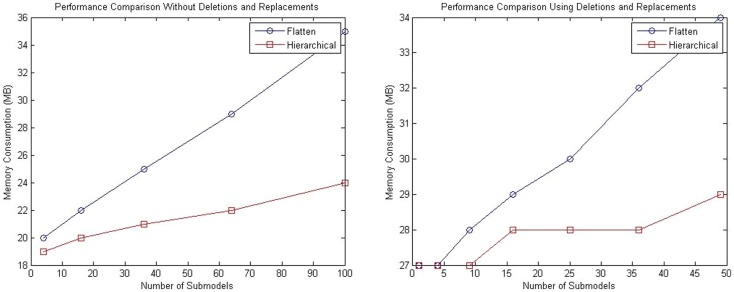
**Comparison of memory consumption of the SSA using flattening and our hierarchical approach**. The plot on the left shows the comparison of memory consumption for a model without replacements and deletions and the right plot shows the results for a model that includes replacements and deletions.

For both tests described in this section, analysis of memory consumption has been performed. The left plot of Figure 12 shows the results for the model with a population of repressilator circuits in sub-models that have no interaction between each other. The model suggests that hSSA takes less space over the long run compared to the flattening approach. Similarly, for the model customized with connections of sub-models using replacements and deletions, hSSA still takes less space as shown in the right plot of plot of Figure 12.

## 9 Discussion

This paper proposes hSSA, a hierarchical simulation method for the analysis of genetic circuits. This method is intended for the analysis of dynamic systems. Results have shown that the extra time spent in the preamble stage of the simulation of a hierarchical model is substantially reduced by not flattening out the hierarchy while the simulation time is essentially equivalent. This fact might be counterintuitive since the hierarchical simulator has to perform replacements and deletions on the fly. However, the time spent on this task is more than compensated by the fact that the hierarchical method avoids flattening and deals with smaller data structures. The total simulation time for the hierarchical simulator grows at a nearly linear increase with respect to model size, whereas the simulation time for a flat simulation has an exponential rate.

**Figure 13:**
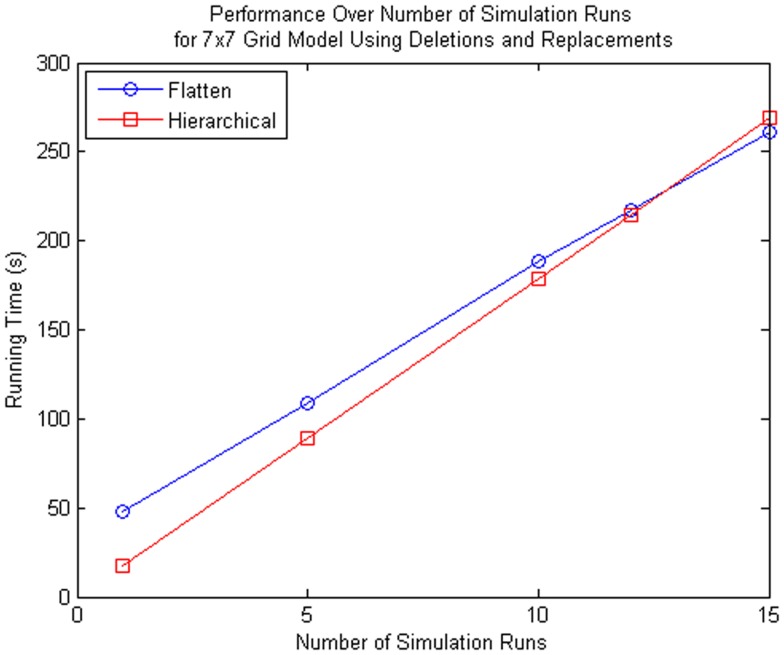
**Comparison of performance for many simulation runs of the SSA using flattening and our hierarchical approach for the 7 × 7 grid model that includes replacements and deletions**. Note that the time spent on flattening is compensated when you do multiple runs since the hierarchical simulation method has some overhead when there are custom connections between top-level model and sub-models.

Even though the proposed method is more inefficient for multiple simulation runs as shown in Figure 13, the proposed method is beneficial to some particular applications. Using the 7 × 7 grid model we compared the performance of the hierarchical method and the method that flattens out the hierarchy, we see that if you perform more than 12 simulation runs, the method with flattening is better. However, the hierarchical simulator is intended for single runs, where the user is interested to see whether the model is behaving as expected. If the user wants to see the average behavior of the model and perform multiple runs, then it is preferable for the user to simulate using hSSA to ensure the model of interest is meeting the expected requirements do before performing multiple runs using the flattening method. Furthermore, population models, which this method aims for, does not require multiple runs since the stochastic nature of each cell in the population is captured by each sub-model, where the average behavior of the population model is the average of each cell.

One of the limitations of the proposed simulator is that it supports only two-levels of hierarchy since sub-models are flattened out. Although the algorithm can be applied to many levels of hierarchy recursively, limiting the hierarchy to two-levels is chosen because population models have regular constructs enclosed in sub-models. The proposed algorithm only needs to flatten one of each sub-model, and this operation is not as complex as flattening out the entire model.

While the hierarchical simulator is promising, there are many extensions that can be implemented in the future. The next step is to support dynamic events to model cell division and death, which add or remove models from the simulation dynamically. We feel that the hierarchical simulator is better suited to such changing model structures, and this requirement is actually a major motivation for the development of this simulator. Another future enhancement is dynamic model abstraction. In previous work, significant improvements in analysis time are achieved by removing unimportant details using automated model abstraction before simulation, which improves simulation time while still delivering accurate results (Kuwahara et al., 2006; Madsen et al., 2012b). A dynamic hierarchical simulator has the potential to allow these abstractions to be performed on the fly to manage complexity as needed to balance computational cost with accuracy. Finally, given that dynamic hierarchical models are inherently concurrent, parallel processing can also be explored to further improve simulation time.

## Author Contributions

All the authors in this paper contributed equally to the work.

## Conflict of Interest Statement

The authors declare that the research was conducted in the absence of any commercial or financial relationships that could be construed as a potential conflict of interest.
